# Assessment of Microcirculatory Changes in Patients With Cervical Spinal Cord Injuries and Neurogenic Shock During the Acute Phase Using Near-Infrared Spectroscopy

**DOI:** 10.7759/cureus.77232

**Published:** 2025-01-10

**Authors:** Panu Boontoterm, Siraruj Sakoolnamarka, Karanarak Urasyanandana, Peera Naklaor, Pusit Fuengfoo

**Affiliations:** 1 Neurological Surgery, Phramongkutklao Hospital, Bangkok, THA; 2 Surgery, Phramongkutklao Hospital, Bangkok, THA

**Keywords:** mortality outcomes, near-infrared spectroscopy, peripheral microcirculation, shock in trauma, spinal cord injury, traumatic cervical spine injury

## Abstract

Background

Cervical spinal cord injury (SCI) is a severe condition that can lead to neurogenic shock, a life-threatening complication. Neurogenic shock is characterized by a sudden impairment of sympathetic tone, resulting in vasodilation, hypotension, and bradycardia. This disruption can significantly affect blood flow dynamics, particularly in the microcirculation. Near-infrared spectroscopy (NIRS) is a device that enables the monitoring of tissue oxygenation and the assessment of microcirculatory status. This study aimed to apply NIRS in conjunction with the vascular occlusion test (VOT) to evaluate microcirculatory function in SCI patients with neurogenic shock and analyze its association with in-hospital mortality.

Methods

This prospective study included cervical SCI patients with neurogenic shock on whom NIRS was performed along with VOT within 24 hours after admission in the ICU (T0). Follow-up measurements were taken at the time of the acute phase (D0), and on days 3, 5, and 7. The de-oxygenation (DeO2) slope, re-oxygenation (ReO2) slope, and the reperfusion area were recorded. The prevalence of microcirculatory dysfunction, and mortality rate were primary outcomes of this study.

Results

The prevalence of microcirculatory alterations was 239 (92%), with 122 (47%) of patients still exhibiting alterations at day 7 (D7). Survivors had higher NIRS parameters at D0 compared to non-survivors. The ReO2 slope at D0 was significantly decreased in patients who developed new-onset kidney injury and nosocomial infections.

Conclusion

The prevalence of microcirculatory disturbance was high in patients with an SCI and neurogenic shock, and it was linked to in-hospital mortality and complications.

## Introduction

Cervical spinal cord injury (SCI) is a severe condition that can lead to neurogenic shock, a life-threatening complication. Neurogenic shock is defined as a sudden impairment of sympathetic tone, resulting in vasodilation, hypotension, and bradycardia. This disrupts blood flow dynamics, particularly in the microcirculation. Understanding these changes is crucial for effective management, as tissue perfusion and oxygen delivery are compromised [1,2]. Near-infrared spectroscopy (NIRS) is a non-invasive, real-time tool for monitoring tissue oxygenation and microcirculatory status, making it a promising method for assessing microcirculatory alterations in SCI patients with neurogenic shock. The microcirculatory system is defined as the circulation of blood through the smallest vessels, including arterioles, capillaries, and venules. In neurogenic shock, disruption of the sympathetic nervous system causes vasodilation, shifting of blood to pool in peripheral tissues, reducing central blood volume, and impairing microcirculatory flow. This results in inadequate tissue perfusion, leading to potential organ dysfunction and poor outcomes. Monitoring microcirculation in such patients is essential for timely interventions such as fluid resuscitation and vasopressor support. SCI with neurogenic shock is associated with rapid deterioration and increases mortality rates [3]. The pathophysiology of SCI with neurogenic shock is linked to alterations in blood flow at the tissue microcirculation, oxygenation, and organ dysfunction, often resulting in death. The characteristic maldistribution of blood in SCI with neurogenic shock includes capillary blood flow stagnation and reduced functional capillary density, making conventional hemodynamic measurements insufficient to assess microcirculatory status [4]. During recovery, the function of the systemic circulation may return to near-normal [5], but microcirculatory alterations persist and are difficult to verify with conventional monitoring systems. SCI with neurogenic shock induces complex alterations in oxygen transport and utilization, leading to microcirculatory dysfunction, tissue hypoxia, and organ failure [6]. The pathophysiology of microcirculatory dysfunction in SCI with neurogenic shock involves several mechanisms, such as the functional impairment of various cell types, including endothelial basement membrane cells, vascular smooth muscle cells, leukocytes, and platelets. Decreased capillary density and increased maldistribution have been described in neurogenic shock. NIRS is a non-invasive method for indirectly evaluating microcirculatory systems, often assessed along with vascular occlusion tests (VOTs) to assess oxygen consumption and endothelial function. The thenar muscle is a reliable site for tissue oxygen measurements, as it is less affected by local factors like edema and fat tissue thickness [7-10]. The VOT involves inflating a pneumatic cuff on the arm above the systolic arterial pressure to induce ischemia in the thenar muscles, leading to alterations in tissue oxygen saturation (StO2). VOT is conducted by occluding the artery system proximal to the StO2 probe until a predefined ischemic threshold is reached, after which the occlusion is released, generating dynamic StO2 parameters. Following vascular occlusion, an early de-oxygenation (DeO2) slope is demonstrated, which reflects local oxygen extraction. Upon release of the occlusion, a re-oxygenation (ReO2) slope is seen, determining endothelial function as it depends on tissue perfusion and microcirculatory recruitment after neurogenic shock. The StO2 then increases to a maximum level before slowly returning to baseline, creating the hyperemic response curve. The area under the curve following the ReO2 slope, known as the reperfusion area, serves as an index of microvascular reactivity [11]. The combination of NIRS and VOT provides a dynamic approach to assessing microcirculatory function, with specific alterations in these measurements demonstrated in SCI patients with neurogenic shock. The most sensitive alteration is the ReO2 slope following hypoxic stagnation. In cervical spinal cord injury associated with neurogenic shock, the value of DeO2 and ReO2 slopes are lower than those in healthy individuals, and the reperfusion area is decreased. In critically ill patients, NIRS combined with VOT can help assess tissue oxygen extraction and microvascular reactivity, potentially predicting mortality. In SCI with neurogenic shock, survivors had a significantly higher ReO2 slope as compared to non-survivors, and this relationship remained significant in SCI with neurogenic shock [12]. This study aims to identify microcirculatory alterations in SCI patients with neurogenic shock using NIRS in conjunction with the arterial occlusion test and to analyze the association of these alterations with in-hospital mortality and organ failure.

## Materials and methods

Study design

This prospective observational study involved patients hospitalized in the Trauma Intensive Care Unit (TICU), diagnosed with SCI complicated by neurogenic shock. Neurogenic shock is characterized by a sudden loss of sympathetic tone, resulting in vasodilation, hypotension, and bradycardia.

Study Population

The study included all consecutive cervical SCI patients with neurogenic shock admitted to the TICU between January 2022 and November 2024. Written informed consent was obtained from the patient's legal representatives following study approval. Inclusion criteria included patients aged 20 years or older with cervical SCI and neurogenic shock, who were admitted to the TICU within 24 hours and who had recovered from neurogenic shock (defined as the discontinuation of all vasopressors while maintaining a mean arterial pressure (MAP) of ≥ 65 mmHg for 24 hours). Exclusion criteria included pregnant women, brain-dead patients, non-resuscitated patients, patients with hypothermia (body temperature < 36°C), BMI > 35 kg/m², post-cardiac arrest, any extremity injury preventing NIRS probe placement, contraindications to non-invasive blood pressure measurement, patients with metabolic complications due to cervical SCI, and those who declined to participate.

Study Measures

Upon enrollment, baseline characteristics, including age, gender, BMI, vital signs, comorbidities, and vasopressor use (including maximum dose and duration), were recorded. The sequential organ failure assessment (SOFA) score and arterial lactate levels were also recorded at T0 (study initiation) and D0 (first day of acute phase). NIRS with a VOT was performed within 24 hours of hospitalization (T0), and then on D0, D3, D5, and D7. The Nonin Medical SenSmart™ Model X-100 Universal Oximetry System, along with Nonin's EQUANOX Advance Model 8004CB 4-wavelength sensors (depth 12.5 mm), was used to monitor StO2 during the arterial occlusion test on the thenar muscle (Nonin Medical Inc, Plymouth, MN, US). The VOT was conducted by inflating a pneumatic cuff 50 mmHg above the patient's systolic blood pressure for 3 minutes [13,14]. This induced a progressive fall in StO2, defined as the DeO2 slope. After conducting the pneumatic cuff occlusion at the arterial site for 3 minutes, the cuff was deflated, measuring a ReO2 slope as the StO2 up to a peak level, then gradually declined back to baseline, forming the hyperemic response curve. Key hemodynamics values composed of DeO2 slope, ReO2 slope, and reperfusion area, were recorded and calculated. These data were exported and analyzed offline using Microsoft Excel 365 (Microsoft Corporation, Redmond, WA, US). Outcomes of interest were in-hospital mortality and in-hospital complications, including new-onset acute kidney injury (AKI), based on KDIGO (Kidney Disease Improving Global Outcomes) clinical practice guidelines [15], nosocomial infections after recovery from SCI with neurogenic shock, including pathogen-proven infections that required antibiotic treatment. These infections included central line-associated bloodstream infection (CLABSI) [16], catheter-associated urinary tract infection (CAUTI) [17], and hospital-acquired or ventilator-associated pneumonia [18] and delirium, defined using the Confusion Assessment Method for the Intensive Care Unit (CAM-ICU) [19]. The definition of AKI was demonstrated as an increase in serum creatinine by ≥ 0.3 mg/dl within 48 hours. We used a meta-analysis to estimate the cut-off value for identifying microcirculatory alterations in SCI with neurogenic shock recovery. Based on comparisons of ReO2 between survivors and non-survivors, a significant difference was observed (3.46% (0.60) vs. 2.13% (0.66), p = 0.050). Therefore, a ReO2 value lower than 3.46%/sec was chosen as the threshold for identifying patients with ongoing microcirculatory alterations from cervical SCI with neurogenic shock.

Sample Size

Sample size calculation was based on a prior study published in Critical Care [20], which correlated NIRS monitoring with organ function during ICU care. Assuming a 50% reduction in overall complications with NIRS monitoring, a β error of 0.2, and statistical significance set at p < 0.05, a minimum sample size of 100 patients was required [12-14]. A total of 260 patients in the TICU underwent continuous monitoring of invasive arterial blood pressure, peripheral oxygen saturation (SpO₂), and electrocardiograms.

Ethics Statement

This prospective observational study was approved by the Institutional Review Board of the Royal Thai Army Medical Department on December 15, 2021 (research no. S069h/64). The study adhered to the guidelines set by the Council for International Organizations of Medical Sciences (CIOMS, 2012) and the Good Clinical Practice guidelines of the International Conference on Harmonization (ICH), in accordance with IRBRTA 1818/2564.

Statistical Analysis

Statistical analysis was performed using STATA version 18 (StataCorp LLC, College Station, TX, US). Normality was assessed using the Kolmogorov-Smirnov test. Continuous variables were expressed as means ± standard deviations or medians (25th-75th percentiles) depending on the distribution. Demographic and clinical characteristics between study groups were compared using the student’s t-test or the Mann-Whitney U test for continuous variables, and the chi-square test for categorical variables. A p-value of < 0.05 was demonstrated as statistically significant.

## Results

**Figure 1:**
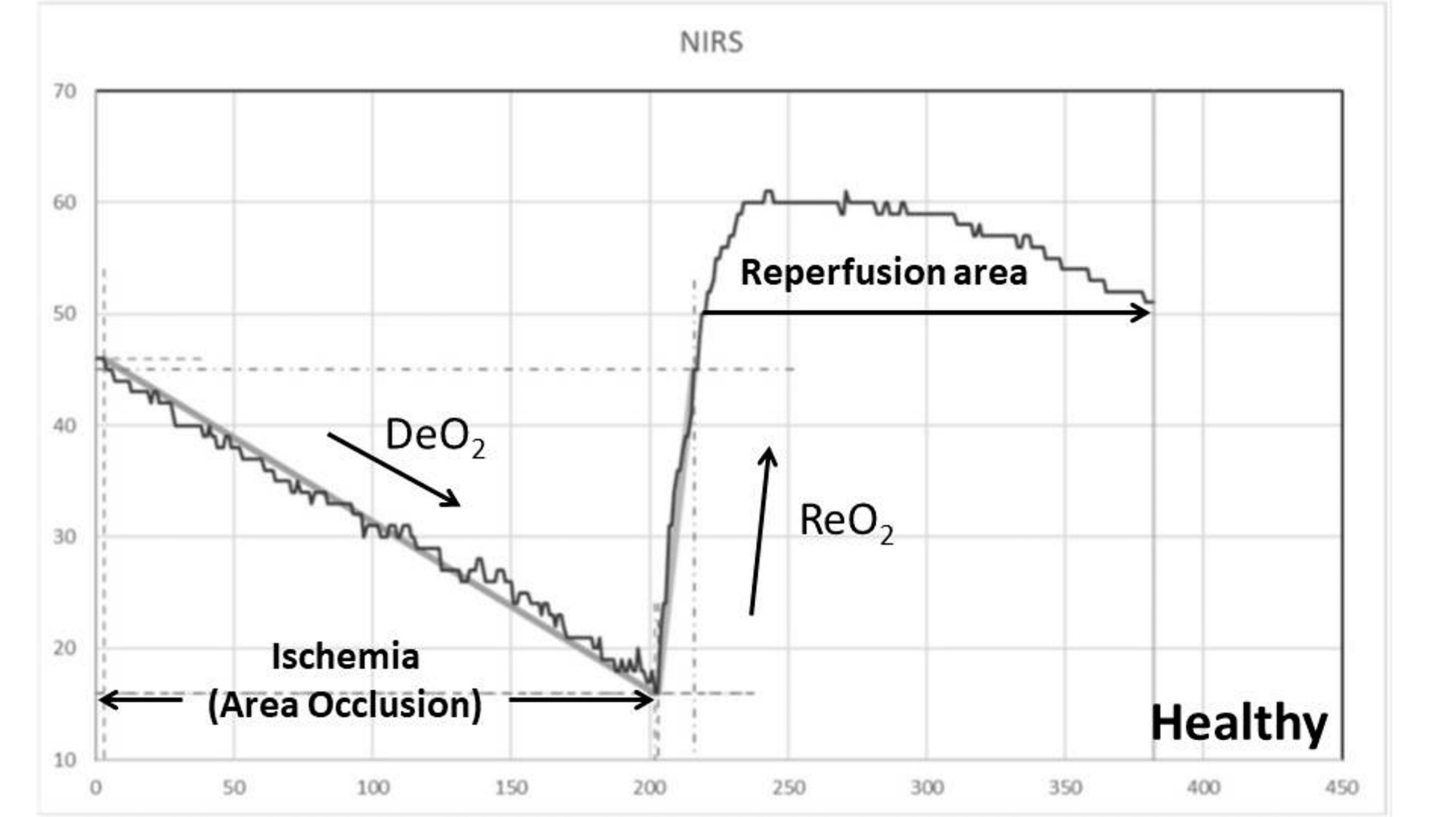
Responses during the arterial occlusion test are typically observed in healthy subjects The responses observed during the arterial occlusion test are from a healthy subject.

**Figure 2:**
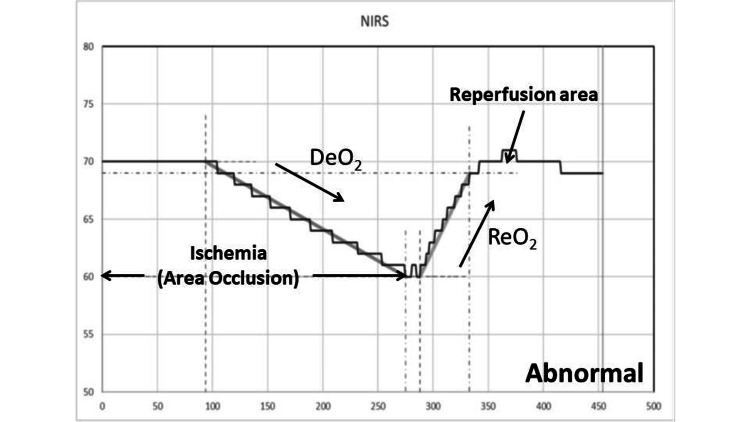
The response of a subject with cervical spinal cord injury and neurogenic shock The responses observed during the arterial occlusion test are from subjects with cervical spinal cord injury and neurogenic shock.

The VOT involves applying a pneumatic cuff around the arm and inflating it to a pressure above the systolic arterial pressure to induce ischemia in the thenar muscles, resulting in changes in StO2. During the VOT, arterial occlusion is performed proximal to the StO2 probe until a predefined ischemic threshold is reached. The occlusion is then released, generating dynamic StO2 parameters. Following ischemia, an initial DeO2 slope is observed, which serves as a marker of local oxygen extraction. Once the occlusion is released, a ReO2 slope is seen, reflecting endothelial function. This reoxygenation depends on the restoration of blood flow and capillary recruitment after the induced hypoxia. As the vascular occlusion is released, StO2 increases to its maximal level and then slowly declines to baseline, creating the hyperemic response curve. The area under this curve serves as an index of microvascular reactivity. Figure 1 shows the typical responses observed during the arterial occlusion test in healthy subjects. Figure 2 shows the responses observed in subjects with cervical spinal cord injury and neurogenic shock. Both the DeO2 and ReO2 slopes are lower in these subjects as compared to healthy volunteers. Similarly, the area under the hyperemic response curve (reperfusion area) is smaller in these subjects than in healthy volunteers, as shown in Figure 1. A total of 260 patients were eligible for the study. NIRS with VOT was assessed in all 260 patients at the time of cervical SCI with neurogenic shock (D0), and subsequent evaluations were assessed at D3, D5, and D7. Clinical data for cervical SCI with neurogenic shock patients are presented in Table 1. The mean age of the patients was 56.75 ± 12.73 years. The comorbidities included diabetes (88; 34%), hypertension (91; 35%), a history of myocardial infarction (10; 4%), liver disease (57; 22%), kidney disease (39; 15%), and a history of cancer (78; 30%). The median lactate level within 24 hours after admission to the TICU for cervical SCI with neurogenic shock (T0) was 4.5 mmol/L (interquartile range (IQR) 3.44, 6.41), and at D0, it was 1.73 mmol/L (IQR 1.28, 2.28). The SOFA score at TICU admission was 14.33 ± 5.64. The maximum dose of norepinephrine was 0.2 mcg/kg/min (interquartile range 0.12 to 0.4). The prevalence of microcirculatory alterations in these patients was 239 (92%), with the percentage of patients showing improvement in microcirculation fluctuating from D0 to D7. At D7, 122 (47%) still exhibited microcirculatory alterations. The in-hospital mortality rate was 96 (37%) for cervical SCI with neurogenic shock patients. The mean age showed no statistically significant difference between survivors and non-survivors (55.68 ± 15.06 vs. 55.29 ± 13.87; P = 0.91). Table 2 compares the clinical data between survivors and non-survivors. Among patients with cervical SCI and neurogenic shock, the median lactate level at D0 was higher in non-survivors than in survivors (2.31 mmol/L (IQR 1.66, 3.15) vs. 1.60 mmol/L (IQR 1.20, 1.80); P = 0.02). Table 3 shows the NIRS parameters for cervical SCI with neurogenic shock patients at T0, D0, D3, D5, and D7. Regarding in-hospital mortality, survivors had higher NIRS parameters at D0 compared to non-survivors: DeO2 slope at D0: 0.15 (IQR 0.12, 0.19) vs. 0.11 (IQR 0.09, 0.13), P = 0.023, 95% CI -26.57 to -0.71, ReO2 slope at D0: 1.51 (IQR 0.88, 2.94) vs. 1.03 (IQR 0.64, 1.22), P = 0.014, 95% CI -1.13 to -0.09, reperfusion area at D0: 756 (IQR 478, 1289) vs. 416 (IQR 173, 598), P = 0.0027, 95% CI -0.0032 to -0.00045. The associations between NIRS parameters at D0 and mortality are shown in Table 4. Additionally, the de-oxygenation and re-oxygenation slopes at D0 were significantly lower in patients who developed new-onset AKI, and nosocomial infections (Table 5).

**Table 1:** Clinical data of patients from cervical spinal cord injury with neurogenic shock during the acute phase (n = 260) Results are presented as mean ± SD, median (IQR), and number (percentage). T0a refers to the onset of cervical spinal cord injury with neurogenic shock; D0b indicates within 24 hours after admission for cervical spinal cord injury with neurogenic shock; IV fluidc denotes intravascular fluid received during vasopressor infusion. SBP: systolic blood pressure; DBP: diastolic blood pressure; MAP: mean arterial pressure; SOFA: sequential organ failure assessment score

Variables	Values
Age (year)	56.75 ± 12.73
Gender	
Male	135 (52%)
Body mass index (kg/m2)	21.74 ± 5.37
SBP (mmHg)	85.83 ± 18.41
DBP (mmHg)	40.94 ± 10.61
MAP (mmHg)	50.90 ± 11.58
Underlying disease	
Diabetes	88 (34%)
Hypertension	91 (35%)
History of myocardial infarction	10 (4%)
Smoking	62 (24%)
Liver disease	57 (22%)
Kidney disease	39 (15%)
History of cancerous disease	78 (30%)
Norepinephrine max dose (mcg/kg/min)	0.2 (0.12, 0.4)
Second line vasopressor usage	18 (7%)
Lactate T0^a^ (mmol/L)	4.5 (3.44, 6.41)
Lactate D0^b^ (mmol/L)	1.73 (1.28, 2.28)
SOFA T0^a^	14.33 ± 5.64
SOFA D0^b^	6 (4, 9)
Vasopressor duration (hour)	65 (43, 87)
IV fluid^c^ (ml)	5,478 (3,827, 8,227)
Microcirculation alteration at acute phase	239 (92%)
Microcirculation alteration at day 7	122 (47%)

**Table 2:** Clinical data of cervical spinal cord injury patients with neurogenic shock during the acute phase: a comparison between hospital survivors and non- survivors Results are presented as means ± SD, median (IQR), and number (percent). T0a refers to the onset of cervical spinal cord injury with neurogenic shock; D0b indicates within 24 hours after admission for cervical spinal cord injury with neurogenic shock; IV fluidc denotes the intravascular fluid received during vasopressor infusion. SBP, systolic blood pressure; DBP, diastolic blood pressure; MAP, mean arterial pressure; SOFA score, sequential organ failure assessment score

Variables	Hospital survivors	Hospital non-survivors	P-value
Age (year)	55.68 ± 15.06	55.29 ± 13.87	0.91
Gender			
Male	135 (52%)	125 (48%)	0.92
Body mass index (kg/m2)	22.23 ± 5.22	21.15 ± 4.22	0.38
SBP (mmHg)	85.20 ± 16.62	83.92 ± 21.46	0.71
DBP (mmHg)	40.02 ± 9.92	39.50 ± 11.75	0.36
MAP (mmHg)	55.08 ± 1.70	54.30 ± 2.60	0.47
Underlying disease			
Diabetes	63 (24.39%)	75 (29.17%)	0.57
Hypertension	196 (75.61%)	184 (70.83%)	0.67
History of myocardial infarction	164 (63.41%)	195 (75%)	0.34
Smoking	95 (36.59%)	65 (25%)	0.58
Liver disease	196 (75.61%)	189 (72.83%)	0.67
Kidney disease	170 (65.41%)	189 (73%)	0.34
History of cancerous disease	90 (34.68%)	70 (27%)	0.34
Norepinephrine max dose (mcg/kg/min)	0.14 (0.08, 0.31)	0.27 (0.15, 0.49)	0.042
Second vasopressor usage	11 (4.58%)	27 (10.50%)	0.27
Lactate T0^a^ (mmol/L)	3.81 (2.62, 5.58)	3.58 (2.65, 5.77)	0.82
Lactate D0^b^ (mmol/L)	1.60 (1.20, 1.80)	2.31 (1.66, 3.15)	0.02
SOFA T0^a^	14.10 ± 2.87	16.08 ± 3.28	0.01
SOFA D0^b^	5 (3, 8)	8 (5, 11)	0.01
Vasopressor duration (hour)	48 (29, 73)	58.50 (41.50, 85)	0.09
IV fluid^c^ (ml)	5,168 (3,263, 7,293)	6,296 (4,348, 13,913)	0.15

**Table 3:** NIRS parameters in cervical spinal cord injury patients with neurogenic shock during the acute phase Results are presented as median (IQR). NIRS: near-infrared spectroscopy; IQR: interquartile range

Parameters	T0 (N=260)	D0 (N=260)	D3 (N=164)	D5 (N=140)	D7 (N=132)
De-oxygenation slope (%/sec)	0.09 (0.08, 0.12)	0.1 (0.08, 0.14)	00.13 (0.11, 0.17)	0.11 (0.09, 0.17)	0.14 (0.1, 0.18)
Re-oxygenation slope (%/sec)	1.17 (0.7, 1.84)	1.2 (0.72, 2.67)	1.55 (0.81, 2.16)	1.36 (0.72, 1.83)	1.67 (1.1, 2.19)
Reperfusion area (%/sec)	664 (384, 1095.50)	622.50 (349, 1010)	571.75 (356, 924)	680 (353, 1272)	804 (430, 1005)

**Table 4:** NIRS parameters in cervical spinal cord injury patients with neurogenic shock during the acute phase at D0 in relation to mortality Results are presented as median (IQR). NIRS: near-infrared spectroscopy; IQR: interquartile range

Parameters	Survivors (N = 164)	Non-survivors (N = 96)	P-value	95% CI
In-hospital mortality				
De-oxygenation slope (%/sec)	0.15 (0.12, 0.19)	0.11 (0.09, 0.13)	0.023	-26.57 to -0.71
Re-oxygenation slope (%/sec)	1.51 (0.88, 2.94)	1.03 (0.64, 1.22)	0.014	-1.13 to -0.09
Reperfusion area (%•sec)	756 (478, 1289)	416(173, 598)	0.0027	-0.0032 to -0.00045
90-day mortality				
De-oxygenation slope (%/sec)	0.15 (0.12, 0.18)	0.13 (0.11, 0.16)	0.164	-19.12 to 3.38
Re-oxygenation slope (%/sec)	1.51 (0.88, 2.94)	1.05 (0.69, 1.27)	0.03	-0.89 to 0.0017
Reperfusion area (%•sec)	787 (478, 1289)	425 (171, 693)	0.0091	-0.0023 to -0.00037
ICU mortality				
De-oxygenation slope (%/sec)	0.15 (0.12, 0.19)	0.11 (0.1, 0.12)	0.032	-34.74 to 0.73
Re-oxygenation slope (%/sec)	1.27 (0.7, 2.87)	1.11 (0.85, 1.2)	0.074	-1.21 to 0.16
Reperfusion area (%•sec)	712(352, 1130)	432 (343, 576)	0.273	-0.0024 to 0.00067

**Table 5:** NIRS parameters in cervical spinal cord injury patients with neurogenic shock during the acute phase at D0 in relation to in-hospital complications Results are presented as median (IQR). NIRS: near-infrared spectroscopy; IQR: interquartile range

Parameters	Negative	Positive	P-value	95% CI
New onset of AKI				
De-oxygenation slope (%/sec)	0.15 (0.12, 0.2)	0.09 (0.07, 0.11)	0.0035	-64.19 to -4.93
Re-oxygenation slope (%/sec)	1.29 (0.78, 2.77)	1.09 (0.67, 1.17)	0.026	-2.07 to 0.17
Reperfusion area (%•sec)	623 (380, 1130)	603 (195, 754)	0.185	-0.008 to 0.0009
Nosocomial infection				
De-oxygenation slope (%/sec)	0.15 (0.12, 0.19)	0.11 (0.1, 0.13)	0.043	-29.64 to 0.42
Re-oxygenation slope (%/sec)	1.34 (0.8, 2.87)	1.11 (0.59, 1.25)	0.021	-1.27 to 0.012
Reperfusion area (%•sec)	693(350, 1128)	449 (343, 714)	0.104	-0.005 to 0.0008
Delirium				
De-oxygenation slope (%/sec)	0.12 (0.1, 0.15)	0.14 (0.11, 0.2)	0.253	-5.55 to 21.76
Re-oxygenation slope (%/sec)	1.24 (0.71, 2.69)	1.25 (1.13, 2.96)	0.971	-0.59 to 0.57
Reperfusion area (%•sec)	679 (343, 1075)	483(309, 1003.50)	0.028	-0.003 to 0.005

## Discussion

In patients with SCI, the disruption of sympathetic outflow from the spinal cord impairs the ability to regulate vascular tone and blood flow. This leads to neurogenic shock, which causes profound alterations in both systemic and regional blood pressure, as well as microvascular perfusion. In this context, NIRS can help detect early signs of insufficient oxygen delivery to tissues, particularly in the brain, muscles, and abdominal organs, where blood flow may be redirected during shock. In the acute phase of cervical SCI, peripheral muscle and abdominal organ perfusion can be compromised. NIRS can be used to assess microcirculation in these tissues, providing valuable insights into oxygen delivery and whether there is adequate perfusion to support recovery [21]. In our study, we demonstrated the presence of microcirculatory alterations in patients with cervical SCI and neurogenic shock using NIRS combined with the VOT. The VOT was performed by inflating a pneumatic cuff to a pressure of 50 mmHg above the patient’s systolic blood pressure and maintaining it for 3 minutes. This procedure resulted in a progressive decrease in StO2, known as the DeO2 slope. After three minutes of occlusion, the cuff was rapidly deflated, initiating the ReO2 slope, which increased to a maximal level. The curve then gradually declined to baseline, creating the hyperemic response curve. This alteration was found to be associated with in-hospital mortality and complications after the acute phase. Our findings indicate that all NIRS parameters at the time of cervical SCI with neurogenic shock were associated with in-hospital mortality. However, only the DeO2 and ReO2 at the time of cervical SCI with neurogenic shock were significantly associated with new-onset acute kidney injury (AKI) and nosocomial infections. NIRS monitoring, when combined with VOT, can assess dynamic changes that reflect oxygen consumption (VO2) and vascular reactivity [22]. This clinical application of NIRS remains promising. To our knowledge, our study is the first to assess microcirculatory function during cervical SCI with neurogenic shock. Although there is no universally accepted definition for recovery from neurogenic shock, our study defined recovery as the absence of vasopressor use for 24 hours. During this recovery period, no patients required resumption of vasopressors, and arterial lactate levels did not increase. The persistence of microcirculatory alteration in cervical SCI with neurogenic shock suggests an independence between macro- and microcirculation. Our findings also suggest that microcirculation may improve later than macrocirculation. Endothelial dysfunction is a key microvascular problem in cervical SCI with neurogenic shock [23]. However, data on the rate of endothelial cell repair in this context is scarce. Previous studies have shown significant endothelial cell repair post-cardiopulmonary resuscitation around the third day, and we hypothesize that recovery from cervical SCI with neurogenic shock may take more time for similar repair processes. Glycocalyx degradation plays an essential role in microcirculatory dysfunction, and although the restoration rate of the glycocalyx in cervical SCI with neurogenic shock recovery is not well understood, it may take several days to complete. Currently, there is no definitive value to indicate microcirculatory alteration. Based on a previous meta-analysis, we used a cut-off point of a re-oxygenation slope of 3.46%/sec [24], as observed in survivors, to categorize patients with remaining microcirculatory alterations after recovery from cervical SCI with neurogenic shock. With a high prevalence of microcirculatory alteration in this study (89.23%), this rate may differ across various populations [25-27]. It is reasonable to infer that persistent microcirculatory alterations contribute to in-hospital complications. An impaired re-oxygenation slope represents the degree of microcirculatory derangement, which is associated with unfavorable outcomes and predisposes patients to future organ dysfunction such as AKI [28]. Pre-existing endothelial dysfunction may increase the risk of infection and lead to new-onset hospital-acquired sepsis [29,30]. NIRS provides a non-invasive means of continuously monitoring tissue oxygenation, which is particularly beneficial for critically ill patients. This allows clinicians to make real-time decisions regarding fluid resuscitation, vasopressors, and other treatments. Since neurogenic shock leads to abnormal microcirculatory dynamics, NIRS offers insights into microvascular oxygen delivery that might be missed by traditional systemic monitoring methods. The continuous, real-time data from NIRS is particularly valuable during the acute phase of cervical SCI with neurogenic shock. It enables immediate feedback on the effectiveness of interventions aimed at restoring microcirculatory flow. Early detection of changes in tissue oxygenation during the shock state allows for prompt interventions, potentially preventing further complications such as organ failure. NIRS data can guide therapeutic interventions, such as fluid resuscitation, vasopressor administration, and other measures aimed at improving tissue oxygenation, ultimately optimizing outcomes for SCI patients in the acute phase of neurogenic shock. The VOT is commonly used to assess tissue oxygenation, microcirculatory function, and the effect of interventions such as ischemia or vasopressor use. However, there are several potential disadvantages and limitations associated with this test.

Discomfort or pain for patients

The pneumatic cuff used to occlude blood flow can cause discomfort or even pain, especially if inflated for longer durations. Some patients may find the pressure uncomfortable, leading to distress, particularly in vulnerable populations like the elderly or those in intensive care.

Potential for hemodynamic disturbance

Prolonged or excessive inflation of the cuff may lead to transient changes in blood pressure and hemodynamic stability, especially in patients with cardiovascular conditions. This can sometimes result in undesirable fluctuations in vital signs.

Risk of tissue damage

If the cuff pressure is too high or applied for too long, there is a risk of causing ischemic damage to the tissue. Although the typical duration is short, any prolonged lack of blood flow can result in cellular injury, particularly in patients with compromised circulation or fragile tissue. Limited in patients with peripheral vascular disease and in patients with peripheral arterial disease or other circulatory issues, the vascular occlusion test may not provide accurate or reliable results. The inability to occlude and then restore blood flow may lead to skewed data or be difficult to interpret. Not suitable for all patient populations, some patients may be contraindicated for this test, such as those with conditions like deep vein thrombosis (DVT), severe arterial occlusion, or other circulatory pathologies. In these cases, the test could exacerbate existing conditions or cause further complications.

Technical variability

The accuracy of the results depends on the precise application of the test, including proper cuff inflation, timing, and monitoring of the affected area. Small errors in technique could result in unreliable or inconsistent measurements of tissue oxygen saturation (StO2). Limited by the cuff location, the test is often performed at specific sites, such as the forearm or thigh, which may limit its ability to provide comprehensive assessments in certain body areas. The results may not be reflective of overall systemic changes, as it focuses on a localized area.

Interference from other factors

External factors, such as temperature, skin pigmentation, or equipment calibration, can influence the results of the vascular occlusion test, leading to potential inaccuracies. Additionally, certain medications or conditions may affect vascular tone and complicate interpretation.

Limited data on long-term effects

While the VOT is generally considered safe when performed correctly, there is limited research on the long-term effects of repeated tests, especially in critically ill or high-risk patients. While the VOT can provide valuable information about tissue oxygenation and vascular function, it is important to recognize these potential drawbacks. Careful consideration should be given to patient suitability, test conditions, and interpretation of results. It is crucial that the test be performed by trained personnel and that the risks are weighed against the benefits for each patient.

Limitations

The present study has several limitations. First, the lack of a standardized cut-off value to assess microcirculatory alterations in cervical SCI with neurogenic shock may influence the primary outcome. Second, NIRS measurements are prone to a low signal-to-noise ratio, which can be influenced by individual differences in tissue characteristics such as varying adipose tissue thickness. Lastly, the study's small sample size may limit the ability to generalize the findings to the broader population of individuals with cervical SCI and neurogenic shock.

## Conclusions

Near-infrared spectroscopy (NIRS) holds significant promise as a tool for assessing microcirculatory alterations in patients with cervical spinal cord injury (SCI) and neurogenic shock during the acute phase. By providing real-time, non-invasive monitoring of tissue oxygenation and microcirculatory flow, NIRS offers valuable insights into the pathophysiological changes occurring in these patients. This capability can guide clinical decision-making, inform management strategies, and potentially improve patient outcomes. However, further research and refinement of the technology are needed to fully realize its clinical potential and address the challenges associated with its application. The prevalence of microcirculatory alterations was found to be high in patients with cervical SCI and neurogenic shock, and these alterations were associated with in-hospital mortality and complications.
